# Differences in single positive formal thought disorder symptoms between closely matched acute patients with schizophrenia and mania

**DOI:** 10.1007/s00406-021-01263-x

**Published:** 2021-05-07

**Authors:** Tilo Kircher, Frederike Stein, Arne Nagels

**Affiliations:** 1 Department of Psychiatry and Psychotherapy, Philipps University of Marburg, Marburg, Germany; 2 Center for Mind, Brain and Behaviour, University of Marburg, Marburg, Germany; 3 Department of English and Linguistic, Johannes Gutenberg-University Mainz, Mainz, Germany

**Keywords:** Poverty of content of speech, Perseveration, TALD, Formal thought disorder, Schizophrenia, Mania

## Abstract

Formal thought disorders (FTD) are a hallmark diagnostic feature of schizophrenia (SZ) and (bipolar) mania (MA). FTD can be separated into positive (pFTD) and negative dimensions. It is unclear whether there are differences in pFTD on a single symptom level between acutely ill patients with SZ and MA, which cannot be attributed to cognitive impairment. We compared single pFTD symptoms in two groups of acutely ill patients with ICD-10 bipolar mania and schizophrenia, closely matched for age, sex, pFTD TALD score, verbal IQ and neuropsychological test performance (executive function, verbal fluency, attention, and working memory). SZ patients had higher severity of the TALD symptoms “perseverations” and “poverty of content of speech” than those with MA (Mann–Whitney *U*, significant, Bonferroni corrected). Speech in acute SZ patients differs from MA in that it conveys little information and adheres to previously mentioned ideas and topics. Matching for confounding variables, such as IQ and cognition, is important when comparing patients with different diagnoses.

## Introduction

Formal thought disorder (FTD) is a hallmark diagnostic feature of schizophrenia (SZ) and (bipolar) mania (MA; DSM-5, ICD-10) [1]. Previously, FTD has been reported as core symptom in SZ patients [2]. The clinical relevance of FTD is further emphasized by its prognostic features as patients with FTD have a higher risk for inpatient treatment, and stay significantly longer in hospital [3]. FTD is commonly separated into positive and negative symptoms [4–7]. Positive FTD (pFTD) is best represented by derailment and loosening of associations, an increased amount of produced speech, neologisms, and stilted (manneristic) speech phenomena. Negative FTD (nFTD) have been conceptualized as a quantitative deficit in speech and thought production (e.g. poverty of speech, slowed thinking, and blocking). Further, objective and subjective FTD can be distinguished. In the current study, we focus on objective FTD, since this concept has mainly been investigated in the past and entered the diagnostic manuals. Diagnostic specificity of FTD in separating schizophrenia from mania remains an unresolved topic, in particular for acutely ill patients [1, 6, 8–10]. The two major questions in this context are [1] whether pFTD differ between the two diagnostic groups in their overall severity (sum scores) and/or [2] in single items, i.e. which pFTD symptom is more prevalent in one disorder than the other.

A meta-analysis including 14 studies with acute patients used standard psychopathological FTD rating scales to assess quantitative differences in pFTD [6]. It revealed that the overall severity of pFTD (sum score) in acute patients with SZ and MA is similar. In clinically stable (out-) patients, SZ exhibit a larger amount of overall pFTD (sum score) than MA patients (meta-analysis of four original studies [6]).

There are only few studies that examined the kind and quantity of single pFTD symptoms, between SZ and MA. In brief, studies using operationalised diagnostic criteria (DSM III and beyond) and FTD rating scales (TDI, TLC, SAPS) found that patients with SZ use more peculiar words, idiosyncratic expressions and neologisms, while MA patients exhibit more rapid, pressured, vague speech [9, 11–16]. However, (1) diagnostic groups in these studies also differed significantly in the overall amount of FTD severity, so the result might be confounded. (2) The diagnostic groups were not matched for IQ and cognitive performance, both of which influence FTD [17], and patients with SZ perform cognitively worse compared to MA [18]. (3) Often both, acute and remitted patients were included [9, 15, 16]. (4) The TDI partly mixes formal thought and language with aspects of speech content, applying a psychoanalytically driven approach [9, 11, 12, 16].

The only study which included acute patients explicitly matched for level of education found the opposite from the above studies, using the CDI. Here, SZ patients used vague references, i.e. they leave the listener with an amorphous and questionable impression rather than a clearly communicated meaning [19]. This leaves the question open, whether the “peculiar use of expressions” in SZ found in the majority of the above studies is in fact due to neurocognitive differences between diagnostic groups, with MA usually performing above SZ patients [20].

It remains unclear, whether differences in psychopathology are due to differences in cognitive impairments and overall amount of pFTD between SZ and MA. We, therefore, studied two acutely ill patient samples with bipolar mania and schizophrenia, closely matched for age, sex, pFTD (Thought and Language Disorder, TALD) score [7], verbal IQ, and neuropsychological test performance (executive function, verbal fluency, attention, and working memory). We were interested in single symptom differences in pFTD between the two patient groups. Based on the literature [19], we hypothesized higher TALD scores in SZ patients for “poverty of content of speech”.

## Methods

### Participants

Patients were part of a larger sample that has been described in detail earlier [7]. They were recruited and interviewed at the Department of Psychiatry and Psychotherapy, Philipps-University Marburg (in- and outpatients), Department of Forensic Psychiatry, Vitos Haina Forensic Psychiatric Hospital (chronic in- and outpatients) and the Department of Psychiatry, Psychotherapy and Psychosomatics, University of Freiburg (in- and outpatients). From this previous study, we matched a subsample of SZ and MA patients using the “MatchIt Package” [21] in R [22] for age, sex, pFTD (TALD subscale score “objective positive factor”, consisting of 15 symptoms), concretism (proverb interpretation test) [23], verbal IQ and neuropsychological test performance (executive function, verbal fluency, attention, and working memory). This resulted in samples of patients with SZ (ICD-10 F20.x, *n* = 16, f = 5/m = 11) and bipolar mania (ICD-10 F31.0, F31.1, F31.2, *n* = 16, f = 5/m = 11) (see Table 1). Patients received antipsychotic medication and/or mood stabilizers. Procedures were approved by the local ethics committee according to the Declaration of Helsinki. All patients gave written informed consent.

**Table 1:** Descriptive demographic, psychopathological and neuropsychological data (uncorrected *p* values)

	Schizophrenia	Mania	*z*	*p*
Age	37.56 (16.91)	47.94 (17.17)	1.75	n.s
Sex	5 f/ 11 m	5 f/ 11 m		n.s
TALD OP	16.65 (6.8)	15.69 (6.57)	− 0.74	n.s
TALD ON	2.81 (2.34)	0.5 (0.73)	− 3.18	0.004
TALD SP	1.69 (1.62)	1.94 (1.81)	0.35	n.s
TALD SN	7.81 (4.96)	3.44 (4.63)	− 2.94	0.004
SAPS sum	30.56 (18.6)	29.13 (13.92)	0.13	n.s
SANS sum	37.88 (17.88)	10.25 (9.25)	− 4.13	< .001
Verbal IQ	99.56 (15.21)	109.25 (14.13)	1.87	n.s
Executive function (TMT)	125.53 (55.89)	133.93 (52.48)	0.46	n.s
Verbal fluency (semantic)	18.94 (5.4)	21 (6.86)	0.87	n.s
Verbal fluency (lexical)	22.06 (19.14)	33.56 (22.65)	0.48	n.s
Attention	103.25 (37.11)	108.71 (24.89)	0.98	n.s
Working memory (digit span forward)	7.19 (1.91)	7.25 (2.46)	− 0.17	n.s
Working memory (digit span backward)	5.67 (1.68)	5 (1.03)	− 0.78	n.s
Concretism	14 (0.00)	13.44 (2.25)	− 1	n.s

Mean (standard deviation)

*TALD* Thought and language disorder scale, *OP* Objective positive FTD TALD subscale, *ON* Objective negative TALD subscale, *SP* Subjective positive TALD subscale, *SN* Subjective negative TALD subscale [7]), *SAPS* Scale for the assessment of positive symptoms [25], *SANS* Scale for the assessment of negative symptoms [24], *TMT* Trail making test, B version [27]), Concretism (= proverb interpretation test [23])

### FTD assessment and rating procedure

Psychopathology was assessed using the TALD [7], SANS [24], and SAPS scales [25]. The TALD is a comprehensive clinical rating scale covering a wide range of FTD symptoms. To reduce heterogeneity, we were only interested in objective FTD symptoms.

Raters were clinically trained psychiatrists, familiar with the in-depth assessment of psychopathological symptoms. All raters were acquainted with definitions and detailed descriptions of the TALD manual. Three rater training sessions were performed including video training sessions of TALD patient interviews. The rating results were compared and jointly discussed afterwards. For the assessment of final inter-rater reliability, raters independently scored the rating scales directly after a patient was interviewed (SAPS/SANS, TALD). Raters achieved good inter-rater reliabilities (ICC) for TALD (0.80), and SAPS/SANS (0.89). All phenomena for the study were evaluated during a 50-min clinical interview and were scored immediately afterwards. In the first part of the interview, the participant was asked to talk about general issues, e.g. topics of everyday life, and hobbies. This was followed by a semi-structured part, during which particular symptoms were explored in greater detail. During the interviews, patients were given enough time to speak freely for several minutes, when possible, after each question or promptly.

### Neuropsychological assessment

On the day of the interviews, we also assessed neuropsychological test performance: premorbid verbal IQ [26], executive functions [Trail-Making Test (TMT) parts A and B] [27], verbal fluency (semantic and letter) [28], attention (D2 test) [29], working memory (number span (forward and backward) [30] and proverb interpretation [23].

### Statistical analysis

Some of the variables were not normally distributed. Therefore, we used non-parametric Mann–Whitney *U* tests [31] to investigate group differences. We applied the Statistical Package for Social Science (IMB, SPSS), version 22, Armonk, NY. To avoid alpha inflation due to the multiple testing procedure, the α level was set to *p* < 0.003, assuming a type I error of *p* < 0.05 (Bonferroni’s correction). In addition, we investigated the relationship between single pFTD items discriminating SZ and bipolar mania patients and global, objective, positive and negative TALD scores to examine whether they should be categorized as positive or negative FTD using correlational analyses (Kendall's tau [32]). Since patient groups were not matched for negative symptoms, we wanted to rule out potential confounding effects of this syndrome using correlational analyses of single pFTD items and total SANS score.

## Results

Descriptive statistics are summarized in Table 1. Groups were well matched for age, sex, pFTD (TALD score “objective positive factor”), SAPS, estimated verbal IQ and neuropsychological test performance, but not for negative symptoms (SANS), where SZ patients displayed higher scores.

We were interested in the TALD pFTD item level differences between patients with SZ vs. MA. The groups differed in the severity of “perseverations” and “poverty of content of speech”, differences in “restricted thinking” did not survive Bonferroni correction, with SZ patients having higher scores as compared to MA patients. For other objective positive FTD symptoms, we found no significant differences between groups (see Table 2). In an exploratory analysis, we did not find Bonferroni corrected differences in nFTD symptoms between groups.

**Table 2:** Objective positive and negative FTD TALD symptoms in groups of patients with acute schizophrenia or bipolar mania

		Schizophrenia (M, SD)	Mania (M, SD)	*z*	*p*
pFTD symptoms	Poverty of content of speech	1.44 (1.03)	0.13 (0.5)	− 3.75	**0.0006**^**a**^
	Perseveration	1.0 (0.97)	0.06 (0.25)	− 3.12	**0.002**^**a**^
	Restricted thinking	1.56 (1.21)	0.44 (0.81)	− 2.72	0.007
	Dissociation of thinking	0.88 (0.89)	0.56 (0.89)	− 1.12	n.s
	Crosstalk	1.44 (0.89)	1.06 (1.06)	− 1.05	n.s
	Circumstantiality	1.94 (1.29)	2.56 (0.63)	− 0.98	n.s
	Phonemic paraphasia	0.38 (0.62)	0.38 (0.81)	− 0.49	n.s
	Semantic paraphasia	0.63 (0.89)	0.63 (0.96)	− 0.14	n.s
	Manneristic speech	0.38 (0.62)	0.56 (0.89)	0.35	n.s
	Rupture of thought	0.94 (0.99)	1.13 (1.03)	0.51	n.s
	Neologisms	0.06 (0.25)	0.25 (0.58)	1.08	n.s
	Tangentiality	1.88 (0.89)	2.19 (0.98)	1.31	n.s
	Derailment	1.81 (0.83)	2.19 (0.83)	1.43	n.s
	Pressured speech	0.81 (1.05)	1.38 (0.89)	1.67	n.s
	Logorrhoea	1.44 (1.21)	2.19 (0.83)	1.78	n.s
nFTD symptoms	Poverty of speech	0.94 (1.12)	0.00 (0.00)	− 2.94	0.035
	Slowed thinking	0.56 (0.89)	0.00 (0.00)	− 2.93	n.s
	Concretism	1.31 (1.14)	0.5 (0.73)	− 2.14	0.047

Uncorrected *p* values

^a^Indicates significant difference after Bonferroni correction for multiple testing (*p* < 0.003)

Next, we examined whether single pFTD items discriminating SZ and bipolar mania should be categorized as positive or negative FTD. Correlational analyses of perseverations and poverty of content of speech with the positive (TALD OP) and negative (TALD ON) score of the TALD were performed. Poverty of content of speech was positively correlated with both the sum score of negative (TALD ON) (*r*_*T*_ = 0.43, *p* = 0.0005) and positive (TALD OP) (*r*_*T*_ = 0.41, *p* = 0.0005) FTD. In addition, perseverations were positively correlated with the positive (TALD OP) (*r*_*T*_ = 0.34, *p* = 0.021) and negative (TALD ON) (*r*_*T*_ = 0.33, *p* = 0.033) FTD score, too. Furthermore, we correlated preservations and poverty of content of speech with the SANS and SAPS sum scores, again to see whether these symptoms are rather related to overall positive or negative symptoms. Poverty of content of speech was positively correlated with the sum of all negative symptoms (SANS) (*r*_*T*_ = 0.53, *p* = 0.0002) but not with the sum of all positive symptoms (SAPS) (*r*_*T*_ = 0.23, *p* = 0.128). Perseverations were positively correlated with the sum of all negative symptoms (SANS) (*r*_*T*_ = 0.38, *p* = 0.009) but not with all positive symptoms (SAPS) (*r*_*T*_ = 0.28, *p* = 0.059).

## Discussion

We examined the differences of single pFTD symptoms in acute patients with schizophrenia and bipolar mania. The two groups were matched for the total amount of pFTD, verbal IQ and neurocognitive performance, as well as for age and sex. Patients with SZ scored higher in the TALD symptoms “poverty of content of speech” and “perseveration”, i.e. their speech was adequate in amount, but it conveyed little information and adhered to previously mentioned ideas and topics.

A recent study examining single symptom pFTD differences, found more “deviant verbalisations” in SZ as compared to MA patients [9]. In the TDI used in this study, “deviant verbalisations” is a syndrome or factor comprising the symptoms and sub-symptoms “peculiar verbalization and response” (peculiar expression, stilted inappropriate expression, idiosyncratic word usage), “queer response” (queer expressions, queer imagery, queer word usage), “absurd response” and “neologism”. Although there is no formal comparison study between the TDI and the TALD, the TDI “deviant verbalisations” would roughly correspond to TALD symptoms “neologisms”, “sematic paraphasia”, “phonemic paraphasia”, “manneristic speech”, “clanging”, and to some extent also “dissociation of thinking”. This finding is similarly reflected in other studies using the TDI [9, 11, 12, 16], the TLC [13, 14] and the SAPS. These differences between the above and our study may be explained by their methodological dissimilarities: (1) whereas we examined acute patients, others only included remitted or both patient groups [9, 15, 16]. We know from previous FTD studies that only remitted, but not acute SZ vs. MA patients differ in the overall amount of pFTD [6]. (2) Therefore, we matched groups for pFTD sum score, while there was no pFTD group matching in other studies [9, 11–16, 33]. (3) We further matched groups for verbal IQ and neurocognitive performance as these domains are known to modulate pFTD [17]. We were not interested in the relation of cognitive performance with symptomatology, but treated this as a (nuisance) variable of no interest, therefore, our matching for cognitive performance between SZ and BD. If we did not match for cognition, we could not tell apart the effect of diagnosis from cognitive performance. (4) In addition, we restricted our sample to SZ patients while some of the above studies combined SZ and schizoaffective patients into one group. (5) The TALD was scored after a 50 min. clinical interview, whereas the TDI/TLI is applied to short responses to Rorschach inkblots or pictures fromthe thematic apperception test [9, 11, 12, 16, 33]. Hence, the amount of longer speech responses is different in the two settings. This difference in the assessment procedure leads to a higher amount of shorter expressions using the TDI reflected in the “deviant verbalization” group difference. In contrast, the TALD additionally scores deviances integrated across somewhat longer time periods (e.g. loosening of associations), echoed in our finding of group differences in more global speech items such as poverty of content of speech. (6) Lastly, the TALD is the rating of a clinical interview while the TDI/TLI are rated on the basis of verbatim transcripts.

Whereas our study found that SZ patients spoke at some length but did not give adequate information to answer a question or bring about a story, the majority of previous studies showed this speech pattern for MA, with SZ patients in contrast using inadequate, idiosyncratic words or short expressions. This might be due to our small sample size. But in line with our findings, the only other study which included acute patients matched for level of education applying the CDI, found SZ patients using vague references, i.e. they leave the listener with an amorphous and questionable impression rather than a clearly communicated meaning [19]. Our finding corroborates these and similar results of an older study, where MA and SZ score comparably in their IQ [4]. In summary, we conclude that the idiosyncratic word usage in SZ patients found in most previous studies is due to lower neurocognitive performance and/or IQ in the SZ (vs. MA) group. If matched for IQ and cognitive performance, acute SZ emerges as the disorder with higher levels of poverty of content of speech.

Previously, studies [33, 34] investigating linguistic features in SZ and BD patients revealed disorganization being characterized by an excessive use of connectives in SZ. This finding corroborates our results since one might assume patients presenting a high amount of poverty of content of speech and using many perseverations tend to excessively use connectives linking utterances lacking in content.

Other studies, using the TLC, classified poverty of content to be an aspect of negative thought disorder [35], and the status of perseveration as either positive or negative FTD is somewhat ambiguous. In our study, poverty of content of speech was correlated equally with both the positive as well as the negative TALD FTD subscale. Therefore, a precise assignment to one of the two sub-scales is not possible. Moreover, poverty of content of speech and perseverations were correlated with the sum score of negative symptoms (SANS). How do we interpret these findings? (1) If we stay strictly with the data our two TALD symptoms in question, perseverations and poverty of content of speech, have been categorized into “objective positive FTD” by our original factor analysis, comprising *n* = 210 patients [7], using the definitions of the TALD scale. Therefore, within the TALD framework, the issue is clear, perseverations and poverty of content of speech are classified as positive FTD. (2) In our current small sample of *n* = 32 patients, perseverations and poverty of content of speech both correlate with each, the positive and the negative TALD FTD score. Therefore within the framework of our small study, the issue is unresolved, whether perseverations and poverty of content of speech should be classified as positive or negative FTD. (3) If we now go beyond the TALD and, again within our small study of *n* = 32, correlate perseverations and poverty of content of speech with the SAPS and SANS sum scores, we find correlation coefficients between 0.23 and 0.53, which partly reach significance. Again we would argue, there is no clear picture as to whether perseverations and poverty of content of speech should be categorized as positive or negative (general or FTD) symptoms. Importantly, the definitions of poverty of content of speech and perseveration differ between the TALD and the SAPS/SANS. Consequently, a strict assignment of poverty of content of speech and perseverations to one of the two oversimplistic dimensions positive or negative symptoms is not possible. In the end, it is a conceptual issue, on which of only two FTD or general psychopathology (SAPS/SANS, etc.) factors a single symptom is grouped into. We would think that ideally, FTD should not be reduced to two broadly defined dimensions separating language and thought into something “too much” or “too little” but rather as a complex system of several levels or dimensions of speech abnormalities. In line with this argument and based on previous research, FTD can be divided into one to six factors [36–38]. However, the exact number of FTD depends on the sample, the scale and the statistics used. While studies showed consensus about one negative/poverty domain [39], positive FTD has been divided into two (e.g. disorganization, verbosity) to five (e.g. disorganization, idiosyncratic, semantic, attentional, and referential) factors in SZ patients [37, 38], and there is furthermore a continuing debate on the number of FTD dimensions across disorders. Factor analyses to categorize single FTD symptoms into domains are dependent on the scale, factor analytical method and population used. To sum up, there is an unclear assignment of poverty of content of speech and perseverations into a simplified system of positive or negative FTD (or, more broadly positive/negative general psychopathology) in the literature. We would like to rest our interpretation on our data and the TALD definitions, because this is what our study is based on. Our results are in line with two previous studies that—unintended?—had SZ and MA patients with equivalent education/IQ with a similar result as ours [4, 19].

One limitation of our study is the small sample size (potential type two errors), which is, however, compensated by unique matching of our groups. Moreover, rigorous non-parametric testing and Bonferroni correction was used to explore group differences. TALD raters were also conducting the clinical interview, and thus not blind for the diagnosis, but inter-rater reliability of the TALD [7] was high.

In summary, we found single symptom differences between acute patients with schizophrenia and mania, who were closely matched for potentially confounding variables. SZ patients exhibited more vague speech being characterized by both little content and restricted topics. Our results should give rise to more research into cross-disorder FTD symptoms, particular longitudinally over time. Fine-grained psychopathological phenotyping will inform novel stratification for genotyping and symptom informed neuroimaging research [1].

## Data Availability

The data that support the findings of this study are openly available in https://www.zenodo.org at https://doi.org/10.5281/zenodo.4068329 Reference Number https://doi.org/10.5281/zenodo.4068329.
